# A Response to Coren’s Objections to the Principle of Alternate Possibilities as Sufficient but not Necessary for Moral Responsibility

**DOI:** 10.1007/s11406-017-9819-y

**Published:** 2017-05-25

**Authors:** Garry Young

**Affiliations:** Division of Psychology, Nottingham Trent University, Burton Street, Nottingham, NG1 4BU UK

**Keywords:** Unequivocally and equivocally immoral/moral, Contextualized moral claims, Moral inverse, Twin world congruence

## Abstract

In this paper I respond to Coren’s argument against my 2016 paper in which I present a case for the principle of alternate possibilities as sufficient but not necessary for the ascription of moral responsibility (PAP_(S)_). I concede that Coren has identified aspects of my original position that are vulnerable to counter-examples. Nevertheless, through a simple amendment to my original argument I am able to respond to these counter-examples without undermining the foundations on which my 2016 paper was built. Moreover, it is my contention that the main challenge Coren presents to my original paper involves making explicit that which is already implied within PAP_(S)_. Therefore, while I acknowledge that my argument for PAP_(S)_ requires further clarification, this can be achieved (as I demonstrate here) without undermining my original position.

In Young (2016), I present the principle of alternate possibilities (PAP) as a sufficient but not necessary condition for the ascription of moral responsibility. PAP_(s)_ reads as follows: A person is morally responsible for what they have done if they could have done otherwise. I further stipulate that for the ascription of moral responsibility at least one alternate possibility must be morally praiseworthy or at the very least not immoral. Coren raises two objections to PAP_(s)_. I intend to respond to each in turn, but will focus on Case 1 of Objection 1 which I discuss at length. I will then discuss Case 2, briefly, before moving on to Objection 2, Case 3.

Through Objection 1, Coren seeks to amend PAP_(s)_’s requirement for an alternate possibility – from something that would *appear* to be categorical (i.e., morally praiseworthy or at the very least not immoral in some absolute sense) to something more relative – by arguing that the relationship between what is selected (E) and the alternative(s) (other than E) must be such that E is clearly morally superior or inferior to ‘other than E’, rather than being moral or immoral per se. Thus, the amended PAP_(s)_ (presented as PAP_(s)_*) states:

PAP_(s)_* =_df_ S is morally responsible and thus praiseworthy or blameworthy for E if S could have done something clearly morally superior or clearly morally inferior to E. (Coren 2017, p.6)

In response, I argue that PAP_(s)_* presents more formally a relationships between alternate possibilities that further analysis reveals PAP_(s)_ already accommodates, at least when Young’s (2016) requirement for alternate possibilities is explained in more detail and amended slightly in response to Coren’s comments (this slight amendment also enables PAP_(s)_ to accommodate the objection raised by Case 2 of Objection 1). Therefore, while I accept that PAP_(s)_* provides *some* clarity (although not absolute) which, by comparison, PAP_(s)_ lacks – insofar as I concede that PAP_(s)_ may give the impression that it requires or postulates categorical moral claims, even though it does neither – I do not accept that PAP_(s)_* overcomes alleged problems inherent within PAP_(s)_ because I believe that PAP_(s)_ is able to accommodate the challenges Coren presents in Objection 1, as I intend to show.

At the risk of pre-empting my argument, I believe PAP_(s)_ is able to resist the challenges posed by Objection 1 because I take the fact that PAP_(s)_* stipulates that E must be *clearly* morally superior or *clearly* morally inferiors to mean that PAP_(s)_* employs the same reasoning as PAP_(s)_. To explain: if satisfying PAP_(s)_* requires that E is *clearly* morally superior or inferior to ‘other than E’, then the term ‘clearly’ would seem to denote a difference in kind, rather than degree. On the other hand, if E is meant to differ simply by degree then PAP_(s)_* needs to articulate this requirement in a less ambiguous way (as ‘clearly’ is not clear enough). Distinguishing E from ‘other than E’ is essentially what the original PAP_(s)_ is doing with reference to outcomes that are identified as ‘moral’ *relative* to outcomes that are considered ‘immoral’. Applying these contrasting moral labels is therefore a way of *clearly* demarcating between outcomes whilst also identifying one outcome as morally superior or inferior to another (where this is found to be the case, of course). In short, given that the same reasoning is applied, it is my contention that PAP_(s)_ provides not only a legitimate means of ascribing moral responsibility to S but does so in a manner compatible with PAP_(s)_*.

In Objection 2, Coren argues that PAP_(s)_ lacks the same intuitive appeal as the original PAP. In response, I argue that PAP_(s)_ maintains some of this intuitive appeal but also that our intuition needs to take account of the subtle difference between acknowledging that one could have done otherwise on a given occasion and the role alternate possibilities can play even when one could not have done otherwise on a given occasion. Coren also seeks clarification regarding my use of ‘congruence’ in the Twin World Condition (TWC), which I attempt to provide through, among other things, a response to his Case 3 objection.

## Response to Objection 1

### Case 1

Coren’s first objection is said to demonstrate how PAP_(s)_ ascribes moral responsibility to individuals in cases where we would not intuitively expect it; it is therefore too broad. This objection is presented using two vignettes in the form of Case 1 and Case 2. Case 1 reads as follows:

Case 1: A train is approaching and will continue quickly down one of two tracks. On Track A there are 25 people tied up. On Track B there are 26 people tied up. If S flips a switch conveniently located beside S then the train goes down B. If not, it goes down A. S must choose to either flip the switch or not (mutually exclusive and jointly exhaustive options). S knows all of this and decides to not flip the switch. (Coren 2017, pp.3–4)

According to Case 1, S can do one of two things: either flick the switch (the ‘action’ option), or not flick the switch (the ‘inaction’ option). One consequence of S’s action or inaction (or, if you like, one event that would transpire because of what S does) can be described in terms of the number of people killed. By choosing the ‘action’ option, a group of 26 people would be killed. Conversely, by choosing ‘inaction’, a different group of 25 people would be killed. Importantly, though, either description only *partially* captures what would occur as a consequence of S’s action or inaction. In addition, if S decides to flick the switch (‘action’ option), 25 people are not killed by the train. Likewise, 26 people are not killed if S does nothing (‘inaction’ option). Intuitively, each of these partial outcomes can be given the following moral status (ceteris paribus):

- Allowing 25 people to be killed is immoral
- Allowing 26 people to be killed is immoral
- Not allowing 25 people to be killed is morally good
- Not allowing 26 people to be killed is morally good

Of course, the possible events Case 1 permits do not *just* involve 25 or 26 people being killed (depending on what S allows to happen); nor do they *just* involve 25 or 26 people not being killed. Instead, the event Case 1 actually describes is 25 people being killed alongside 26 people being saved; although it could have described the opposite, should S have chosen otherwise. I have captured each of these co-occurrences below in relation to the two options available to S:

1. Action (flicking the switch): Allows more people to be killed than are saved
2. Inaction (not flicking the switch): Allows more people to be saved than are killed

According to PAP_(s)_, S being able to do otherwise is sufficient for S to be ascribed moral responsibility; and as Coren points out, S is able to do otherwise (i.e., action or inaction). More specifically, S satisfies the following conditions:

- It is possible for S to do otherwise insofar as S is both physically and mentally capable of doing otherwise (including being aware of alternate possibilities that he is able to select/perform).
- All external conditions required to enable alternate possibilities are satisfied.

But, as already noted in Young (2016), I also stipulate the following for the ascription of moral responsibility, which Coren acknowledges:

[T]he notion of ‘alternate possibilities’ should be understood to mean that there is at least one alternate possibility that is considered morally praiseworthy, or at the very least not immoral. (p.964)

With Case 1, it is true that (a) and (b) are satisfied. Nevertheless, do the options available to S allow an outcome that is morally praiseworthy or at the very least not immoral?

(i) S’s action (flicking the switch) allows more people to be killed than are saved. Unequivocally, I would say that this is an immoral outcome (ceteris paribus)

If the two groups differ *only* in terms of their size then allowing the larger group to be killed cannot be justified, morally.

(ii) S’s inaction allows more people to be saved than are killed. The morality of this outcome is, I accept, equivocal.

With (ii), one could say that it is a morally good outcome because more people are saved than are killed; although I accept that one might question how it could be so given the number of people killed (some might view the event as a kind of Pyrrhic victory). That said, the fact that more people are saved than are killed makes it hard to justify (I would argue) classifying the outcome as immoral. Given this, PAP_(s)_’s requirement for alternate possibilities is satisfied: because the possible outcome described in (ii) is not immoral. That said, I shall now attempt to justify why S’s inaction should be seen as allowing a moral outcome, not merely one that is not immoral (a move that is also compatible with the slight amendment I intend to make to my definition of alternate possibilities).

### S’s Inaction Allows a morally Praiseworthy (Qua morally Good or Correct) Outcome

Consider how Coren describes S’s inaction:

…[I]n Case 1, the *best* thing that S could have done is to allow 25 people to be killed: S’s two options were the *only* options (jointly exhaustive of the domain of possibilities). But we probably would not want to praise S for allowing 25 people to be killed by not pulling a switch. Nor, I suspect, would we blame S for doing what was S’s best option available. (Coren 2017, p.4; emphasis in original)

When Coren refers to S’s inaction as the *best* thing he could have done, he is not implying that it was unequivocally the best thing to have done but, rather, that it is the best thing *under the circumstances*. I take this to mean ‘given the alternative’. The alternative, in this case, amounts to an unequivocally immoral outcome: namely, allowing more people to be killed than were saved. Allowing more people to be saved than were killed is therefore a morally superior outcome to the alternative, and this is the point Coren’s is making. I do not disagree with Coren, here. Instead, I wish merely to demonstrate that what Coren makes explicit through PAP_(s)_*, is a relational quality that PAP_(s)_ already endorses, only tacitly so.

To see how, first consider a further comment made by Coren in addition to claiming that S’s inaction was the best thing to do: that it is likely that we would not want to praise S for allowing 25 people to be killed, but nor should we blame him for this. What Coren seems to be suggesting with this comment is that the outcome of S’s inaction is not in and of itself morally praiseworthy, although it is morally superior to the alternative (the outcome of S’s action). Thus, for Coren, the outcome of S’s inaction is not deserving of moral praise but is morally superior to the alternative (an alternative I have argued is unequivocally immoral). Hence, it is the best option available to S.

If this is the case then the two options available to S are as follows: one whose outcome is immoral (the ‘action’ option) and one whose outcome is not morally praiseworthy but is morally superior to the immoral alternative (the ‘inaction’ option), which I will assume raises it above the level of immoral (although see discussion below on a ‘Sophie’s choice’ situation for an example of where this is not the case). Given this, according to PAP_(s)_, S should be ascribed moral responsibility. So how should we judge S? I agree that we should not blame S for choosing inaction. But if we are not to praise him, either (as Coren suggests), then what are we left to do: just shrug our shoulders? If this were the case then PAP_(s)_ would be in trouble, as Coren claims.

What I aim to show is that the outcome of S’s inaction can, and in fact should, be identified as morally praiseworthy (although not unequivocally so), and that the justification for claiming a morally praiseworthy outcome is based on the same ‘relativity’ requirement PAP_(s)_* expresses. In other words, it is my contention that in order to acquire the status of moral praiseworthiness (and justifiably so), the moral status of the outcome of S’s inaction must be contextualized *relative to* the status of the outcome of S’s action (which amounts to ‘other than E’, in this case); and while this relativity is not expressed within the formulation of PAP_(s)_, it is nevertheless *the* means by which the moral status of a particular outcome is determined (although see below for a caveat in the case of unequivocal moral claims).

To be clear on this point, for the ascription of moral responsibility, where an outcome (E) is considered either morally praiseworthy or immoral, there must always be a possible alternate outcome that is not equivalent to this. But the moral status of E does not have to be unequivocally moral or immoral; rather, E need only be moral or immoral relative to the alternate possibility or possibilities available. Where an outcome is judged to be unequivocally moral or immoral then it must be ascribed this status not just relative to the alternate possibilities available but to *all* alternate possibilities, whether available or not. Thus, with regard to Case 1, when I said that (ceteris paribus) allowing more people to be killed than are saved is unequivocally immoral, for this to be so, there must be no possible outcome that this outcome is morally superior to. In the case of S’s inaction, it is my contention that the outcome allowed by S not flicking the switch is not unequivocally moral – because it is not morally superior to *all* alternate possibilities – but it is morally superior to all of the alternatives available.

What I am saying here is not strictly in accordance with the definition of alternate possibilities presented in Young (2016), which I originally formulated (carelessly, I accept) within the context of describing an alternate possibility to an immoral outcome only. Coren presents an example that shows the original definition to be problematic, but not insurmountably so. The amendment I make therefore maintains the spirit of what I originally intended, but is not restricted to describing alternatives to immoral outcomes.

To illustrate Coren’s objection: suppose S does something morally praiseworthy but, as it turns out, all of his options allow a morally praiseworthy outcome. In such a situation, should S be ascribed moral responsibility? After all, he could not have done other than he did, which was to act morally. I concede that the definition of alternate possibilities found in Young (2016) would require that S be ascribed moral responsibility and, given the moral praiseworthiness of each available outcome, be praised for what he did (recall that the original definition requires that at least one alternate possibility be morally praiseworthy or at the very least not immoral; something which is satisfied here). Given the validity of Coren’s objection, I suggest amending the definition of alternate possibilities to the following: At least one alternate possibility must be available that is the moral inverse of whatever else is available. As noted, this amendment retains the spirit of what I intended in the original version (which was discussed in the context of an immoral outcome) but is able to overcome the objection just raised. When defined in this new way, S should not be ascribed moral responsibility.^1^

With this amendment in mind, and in order to justify the claims that I have been making, we need to understand that allowing 25 people to be killed is a partial outcome of S’s inaction. As noted previously, in and of itself, I consider (intuitively) allowing 25 people to be killed an immoral thing to happen. Therefore, if this was the sole consequence of S’s inaction then, of course, S should not be morally praised for allowing such a thing to occur. But this is not the full extent of the outcome we are judging: for this is not the full (immediate) consequence allowed by S’s inaction. The full consequence is that S’s inaction allowed more people to be *saved* than were killed, and this remains the case relative to the only available alternate possibility. It is for this reason that I believe Coren describes S’s inaction as the best thing he could have done (under the circumstances). Now, if it is the best thing he could have done, is it not the *correct* thing to have done (under the circumstances)? If it is the correct thing to have done then, given the moral relevance of the outcome, is it not the *morally* correct thing to have done?

The moral status of an outcome will shift depending on the context in which it is placed: that is, relative to the alternatives. To borrow an example from Coren: suppose S could have diverted the train onto a third track on which no one was tied. As a result, no one was killed. In this context, diverting the train to the third track allows for a morally superior outcome compared to the two other options. That said, S’s inaction, which would allow 25 people to be killed, is still morally superior to diverting the train towards the 26 people on the other track, but in the context we are *now* discussing should deservedly be said to have allowed an immoral outcome given the new alternate outcome available.

Coren equates the original Case 1 (involving only two options) with the following scenario: imaging a driver whose only options are to steer left and kill a pedestrian, steer straight and do the same, or steer right and, again, kill a pedestrian. Given that there is no morally-relevant way of distinguishing between the pedestrians, each outcome is immoral. This, however, is not the case with Case 1, so they are not equivalent examples. According to PAP_(s)_, which includes the amended definition of alternate possibilities introduced earlier, the driver cannot be ascribed moral responsibility. There is, of course, a further means of ascribing moral responsibility to the driver: the Twin World Condition (TWC), which I also introduce in Young (2016).

**TWC**: Where S’s action E in world W_1_ (a world without alternate possibilities, owing to the possibility of intervention) and S’s action E in twin world W_2_ (which differs from W_1_ only insofar as it is a world with alternate possibilities) are congruent then S in either world is morally responsible for E.

The driver in the scenario just described resides in W_1_ (a world without alternate possibilities that are not immoral). Comparing the driver in W_1_ with the equivalent driver in W_2_ who is able to steer down a fourth road without pedestrians (and therefore inhabits a world with at least one alternate possibility that is not immoral), should the driver’s action in W_2_ be congruent with that of W_1_ then the driver should be ascribed morally responsibility and blamed for her action. Where the action of the driver in W_2_ is incongruent with W_1_ (insofar as she selects the only moral option) then the driver should not be ascribed moral responsibility in W_1_. Importantly, then, and to reiterate, Case 1 (involving S and the two options) is not equivalent to the driver in W_1_ because, in Case 1, unlike the driver in W_1_, S has an option available to him that, in the circumstance permitted, is moral, even if not unequivocally so.

By acknowledging that S did the *best* thing, perhaps we need to bite the bullet and accept that S did the morally correct thing because he allowed more people to be saved than were killed. Of course, the 26 people would have remained ‘not killed’ even if S had not been at the scene (given that S did not flick the switch to change the direction of the train). But this does not make the ‘non-killing of 26 people’ a non-event. Given that S was at the scene, it still features as part of one of two possible outcomes against which S should ultimately be judged given that PAP_(s)_’s criteria were met. Thus, if we accept that saving more people than were killed is a morally good thing to have occurred, especially compared to the alternative of killing more than were saved then we have a morally praiseworthy alternative, which is a sufficient requirement for PAP_(s)_. Importantly, though, and without fear of contradiction, one should be able to say S did the morally correct thing while also claiming that the killing of 25 people was not a morally good thing to have happened (certainly we would not want to praise S for *this aspect* of the full event that transpired, as Coren states). That said, doing the morally correct thing (when the full outcome is evaluated) deserves moral praise; although, perhaps in the context we are discussing, the notion of moral praiseworthiness – as in, singing S’s praises for what he did – could, I accept, be construed as somewhat insensitive, given the tragic deaths that also occurred. But this should not detract from the fact that, in doing the best thing possible under the circumstances, S can be said to have done the morally correct thing.

The use of the term ‘moral praiseworthiness’ (or similar) I concede perhaps gives an unwanted sense of heaping praise on someone for their action (or inaction, in S’s case). What I mean by my use of this term, however, which is also the case in Young (2016), is that a particular outcome constitutes a morally good thing to have occurred. In Case 1, where inaction is interpreted as allowing a morally praiseworthy (*qua* morally correct) outcome, and therefore where an alternate possibility (as defined) is available to S, then S should be ascribed moral responsibility in accordance with PAP_(s)_. Where S (whether through action or inaction) allows what is deemed to be the morally correct outcome to occur then S should be morally praised for his action which, minimally, should be to acknowledge that S has done the morally correct thing.

How does what I have just said differ from what is expressed by PAP_(s)_*? There is no *fundamental* difference; and this is my point. In order to establish the moral status of an outcome (E), one must do so relative to the alternate possibilities available. E’s status should therefore be contextualized (its status is established relative to a particular set of circumstances), unless the moral status is unequivocal, of course, in which case E’s moral status must be established relative to all possible outcomes. However, and importantly, judging that E is morally superior to an available alternate possibility does not *necessarily* make it something that one ought to identify as a morally good outcome, as I shall now demonstrate.

Consider a scenario in which S is told that he has to select one of his two children to be killed. If he refuses to choose then both will be killed. In Young (2016) I referred to this as ‘Sophie’s choice’, adding that each available option was immoral and therefore that S could not be ascribed moral responsibility for any of the outcomes that followed. Even if we leave aside the fact that it is immoral to *intentionally* restrict possible outcomes to those in which at least one child dies, where an alternate possibility could have been made available in which no child dies, it is nevertheless informative to examine this example further. Let us call S’s selecting a child to be killed the ‘action’ option, and S’s refusal to do this the ‘inaction’ option. The outcome of the ‘action’ option is that an equal number of children are saved as are killed. In the ‘inaction’ option, both children are killed. This means that inaction allows more children to be killed than are saved: an outcome I have already identified as unequivocally immoral. Now, given a choice between action and inaction, is not the best option ‘action’?

To illustrate: suppose S is told that both of his children are to be killed unless he presses a button in front of him. Pressing the button randomly lights up one of the children, thereby identifying that child as the one to be either saved or killed. Which it is depends on which outcome has been randomly assigned. Beforehand, S knows neither which child the button will light up nor the randomized outcome (kill or not kill); he knows only that as a result of pressing the button one child will be killed and one will be saved. Under the circumstances, which includes the ‘action’ option’s relation to ‘inaction’ (not pressing the button, meaning both will be killed), not only is ‘action’ (pushing the button) morally superior to ‘inaction’ but it is the morally correct thing to do. This is not to say that killing the same number of children as are saved is, unequivocally, a morally good thing to do – because there are alternate possibilities that are morally superior to this – but it is the morally correct thing to do, here, given the options available.

That said, the scenario just described (involving the pressing of a button and randomized killing) is not representative of ‘Sophie’s choice’. In a genuine ‘Sophie’s choice’ situation, there are in fact two decisions to make, perhaps masquerading as one. The first decision is whether one should select ‘action’ or ‘inaction’. Given the choice, I would say that ‘action’ is the morally correct option to select, for the reasons discussed. But that is not the end of the matter. Having rejected ‘inaction’ – owing to its unequivocal immoral status – S has a further choice to make. Yet each remaining option is equivalent to the other: one child lives and one child dies (not an unequivocally good thing, morally). Ceteris paribus, the choice S has left, because there are no alternatives besides that which is unequivocally immoral (i.e., inaction which allows both to be killed) means that any choice S *intentionally* makes (as opposed to tossing a coin, for example, which is not permitted in a genuine ‘Sophie’s choice’) must be based on some non-morally relevant feature of the children. This is unlike the scenario where S chooses to press the button because, in *that* situation, S did not actually select anyone; rather, a *random* selection was made which was not based on any characteristics of the children at all (moral or otherwise). Making a selection which has moral consequences (i.e., kill or save) based on non-morally relevant characteristics is an immoral thing to do (Patridge 2013). Given that this is necessarily the outcome of S’s further intentional choice within the ‘action’ option; and given that the only other alternate possibility is unequivocally immoral (the already rejected ‘inaction’ option), S has no alternate possibility available to him that is not immoral. Now, what is interesting about this example is that the ‘action’ option, in not being unequivocally immoral, is morally superior to the ‘inaction’ option; but, even so, each outcome available within the ‘action’ option can (and should) be justifiably identified as immoral.

Recall PAP(s)*: S is morally responsible and thus praiseworthy or blameworthy for E if S could have done something clearly morally superior or clearly morally inferior to E. (Coren 2017, p.6]

In doing E (*qua* ‘action’), S could have done something morally inferior to E (namely, ‘inaction’). Does this mean S should be ascribed morally responsibility? No, I do not believe so. This is because PAP_(s)_* does not state that E must be morally superior or inferior to ‘other than E’; rather, E has to be *clearly* morally superior or inferior in order for S to be ascribed moral responsibility. Presumably, this means that outcome E (irrespective of which child S selects), in not being *clearly* morally superior to ‘other than E’, ought to be identified as immoral (the same as ‘other than E’). In other words, although the outcomes differ by some indeterminate degree, they do not differ in kind, insofar as each outcome is of the ‘immoral’ kind.^2^ As such, S should not be ascribed moral responsibility. Yet this is the same conclusion as one should draw when applying PAP_(s)_, as no alternate possibility is available that is not immoral (therefore, there is no alternative available that is the moral inverse of the current options). Thus, even though E is morally superior to ‘other than E’, given that it is not *clearly* morally superior and therefore does not achieve the status of ‘moral’, both PAP_(s)_ and PAP_(s)_* would draw the same conclusion: namely, that S in a ‘Sophie’s choice’ situation should not be ascribed moral responsibility. Likewise, in Case 1, if we interpret PAP_(s)_* as identifying ‘inaction’ as being clearly morally superior to ‘action’ then S should be ascribed moral responsibility; and where one equates ‘clearly morally superior’ with morally correct (or praiseworthy), PAP_(s)_ would, again, draw the same conclusion. Of course, one may argue that, in Case 1, S’s inaction is not clearly morally superior to ‘action’. If it is not clearly morally superior, and ‘action’ is unequivocally immoral, then, according to PAP_(s)_*, S should not be ascribed moral responsibility (because both outcomes must be, I contend, immoral). Once again, under this interpretation, PAP_(s)_ would conclude the exact same thing: namely, S should not be ascribed moral responsibility.

### Case 2

I now turn to a further example used to support Objection 1.

Case 2: S has a choice as to whether S brushes S’s teeth for 1 min and 25 s, 1 min and 26 s, 1 min and 27 s, or 1 min and 28 s. S knows all of this and decides to brush S’s teeth for 1 min and 27 s. (Coren 2017, p.4)

In Case 2, Coren applies PAP_(s)_ to the example of brushing one’s teeth for different amounts of time (1 min 25, 26, 27 or 28 s) but also to whether one chooses to use a blue or black pen, or select red or green grapes. In each example, S has at least one alternate possibility that is not immoral; therefore, each scenario enables S to select an option that is not immoral, as required by Young (2016). Prima facie Coren is correct to state that PAP_(s)_ is satisfied in each example and so (according to PAP_(s)_) S should be ascribed moral responsibility. Coren is also correct to assert that ascribing moral responsibility is not warranted on any of the occasions described by Case 2.

The amendment I have already introduced to the definition of alternate possibilities should, however, provide an adequate response to the objection raised by Case 2 (which Coren again uses to support the claim that PAP_(s)_ allows too broad an ascription of moral responsibility). Given that it is now the case that ‘alternate possibilities’ requires that at least one alternative is the moral inverse of whatever else is available, S should not be ascribed moral responsibility for whichever teeth brushing option he selects (or whether he chooses a blue or black pen or red or green grapes), because there is no moral inverse option available. Importantly, though, one could reasonably declare that there are no options available in any of these examples that are moral/immoral, only *amoral*. The notion of a moral inverse is therefore not applicable. But this is no different to saying that, in relation to PAP_(s)_*, E is neither morally superior nor inferior to ‘other than E’. As such, in the same way that, under PAP_(s)_*, one would claim that S should not be ascribed moral responsibility – because E (in being amoral) is not clearly morally superior or inferior to ‘other than E’ (which is also amoral) – so E is not the moral inverse of ‘other than E’, as demanded by the new definition of alternate possibilities associated with PAP_(s)_. Given this, S should likewise not be ascribed moral responsibility.

Case 2 highlights the need to stipulate what in Young (2016) I had merely assumed: that the options under discussion must have moral *aptness*. In other words, alternate possibilities must be of moral relevance insofar as they are capable of being judged moral or immoral in the context in which they are situated.^3^ All of the examples used in relation to Case 2 do not have moral aptness. There is therefore no need to apply PAP_(s)_ or PAP_(s)_* (for that matter) to these situations, even though each would correctly conclude that S should not be ascribed moral responsibility.

## Response to Objection 2

In Objection 2 Coren claims that by no longer requiring that an alternate possibility is necessary for moral responsibility, my proposal in Young (2016) misses the point of the original formulation of PAP. As he states:

It is prima facie extremely plausible to say that a *necessary* condition for S’s moral responsibility for E is that S could have done something clearly better or clearly worse than E. (Coren 2017, p.7; emphasis in original)

While it is true that a lack of alternate possibilities necessitates S’s action, it would be erroneous to conclude from this that having a choice would have caused S to act differently: for there is an important distinction to be made between S doing E only because he had no choice and S doing E even though he had no choice (which, of course, Frankfurt is aware of in his original paper). An alternate possibility means that we do not have to wonder whether S would have acted differently if an alternate (morally inverse) outcome had been available. Its presence therefore prevents us from having to give S the benefit of the doubt. Importantly, though, PAP_(s)_ does not remove the need (the necessity) for an alternate possibility *in some form*; rather, it removes the need to make necessary the clause that S ‘could have done otherwise’ on *this* occasion.

Where S could have done otherwise (in accordance with my amended definition of alternate possibilities) then S should be ascribed moral responsibility. PAP_(s)_, therefore accords with our moral intuition at least in this regard. Where S could not have done otherwise on a given occasion then the TWC provides a further means of assessing whether he should be ascribed moral responsibility by comparing S in W_1_ (where he could not have done otherwise) with S in W_2_ (where he could). The TWC therefore maintains a role for alternate possibilities by comparing what S is able to do on a given occasion with what he would have done *if* an alternate outcome had been available. Through the TWC, one is able to measure the level of congruence between S’s actions in W_1_ and W_2_. Coren seeks clarification on the role of congruence within the TWC. I aim to provide this clarification now.

Coren’s concern over the role of congruence stems from his view that there may be occasions when S does something that is not obviously moral or immoral, as the Case 1 example involving the train *tries* to illustrate (see also Case 3 below). Yet as I have already argued, in Case 1, S is presented with an unequivocally immoral option (‘action’) and a moral option (‘inaction’). The ‘inaction’ option is not unequivocally moral because there are possible alternatives that are morally superior, only unavailable to S. Therefore, S’s inaction is moral *relative to* the context in which it occurs. This being the case, PAP_(s)_ is able to ascribe moral responsibility to S. Therefore, the TWC is not needed. Instead, it is employed only when PAP_(s)_ is not satisfied and so moral responsibility cannot be ascribed based on its criteria.

To illustrate, consider an example where PAP_(s)_ is not satisfied. Recall the driver who must steer her vehicle down one of three roads, where each manoeuvre will result in the vehicle hitting and killing a pedestrian. Under such conditions, the driver has only immoral options available, and therefore PAP_(s)_ is not satisfied. So we must turn to the TWC.

If S steers straight ahead in W_1_ (killing a pedestrian) and does the same in W_2_, even though in W_2_ a fourth road is available free of pedestrians, then S’s action across worlds is congruent. S should be ascribed moral responsibility and blamed in both worlds. If S instead steers right in W_2_ (killing a different pedestrian) then while I accept that the *action* (literally what was done) across worlds is not the same, it nevertheless remains the case that the outcome of each distinct action is congruent, morally, as both actions are immoral. In such a situation, S in both worlds should again be ascribed moral responsibility and blamed. Where S in W_2_ steers down the fourth (pedestrian free) road, not only does her action differ to that of W_1_ but it is incongruent, morally with the W_1_ outcome. It therefore shows that S would have chosen a moral outcome if one had been available. Unlike the previous two examples, in this situation, S should not be ascribed moral responsibility in W_1_ (but should be in W_2_ and praised).

### Case 3

Coren presents a final example to challenge PAP_(s)_, and in particular the coherence of the role played by congruence within the TWC.

Case 3: In W_1_, S does E: S neither donates to a very good charity, X, nor steals from X. In W_1_, S did not have alternate possibilities that were clearly morally superior or inferior to E. InW_2_, S can steal up to ten thousand dollars from X, or S can choose to donate up to ten thousand dollars to X. In W_2_, S does E: S neither donates nor steals. (Coren 2017, p.8)

The first thing to note about Case 3 is that it is not clear what S actually does in W_1_, only what he does not do. It is quite possible that every day of S’s life he does not do the two things described in Case 3. Yet knowing this to be true still leaves us none the wiser about S’s action E in W_1_, even though there are an indeterminate number of things S could be doing at time t_1_ (in W_1_) that are perfectly compatible with him not donating to a very good charity, X, or with him not stealing from it, including being in a permanent vegetative state. All we know is that S lacked morally superior or inferior options to E in W_1_. As such, one is left to wonder about the extent to which whatever S is doing (in conjunction with the things we are told he is not doing) has moral aptness. In the absence of moral aptness, there is no need to employ PAP_(s)_ and therefore the TWC. Nevertheless, let us engage with the point I believe Coren is making with regard to the use of the TWC and the congruence (or lack thereof) between worlds.

Suppose that whenever S is about to decide to do something morally good (e.g., donate a large sum of money to charity) or something morally bad (e.g., steal money from a charity), Black intervenes (using his neurological devise) to prevent S from deciding and hence from actually doing the morally good or bad thing. Given this, S has no alternate possibility available to him that allows an outcome that is the moral inverse of what he actually does (call this situation W_1_); although, to reiterate, one could argue that S’s “going about his business” (whatever that involves), by doing neither morally good or bad things, means that S’s action lacks moral aptness. On the other hand, S going about his business may be considered a morally good thing to do in the context of a morally bad alternative or a morally bad thing to do in the context of a morally good alternative (where these are available in W_2_).^4^ What Coren is concerned with in Case 3, however, when stipulating that S does the same thing in W_2_ as in W_1_ (i.e., action E: neither donating to the charity nor stealing from it), where morally inferior or superior options are available (stealing from or donating to a charity), is whether we should (a) hold S morally responsible for his action and (b) morally praise or blame him for what he does.

Given that S’s action in W_1_ and W_2_ is the same, S should be held morally responsible for his action in both W_1_ and W_2_. This conclusion is consistent with the role played by TWC because, in Case 3, Coren has contrived that at least one alternate possibility is the moral inverse of another (i.e., stealing and not stealing). That said, what remains to be decided is whether S should be morally praised or blamed for doing what he did (action E), which amounts neither to stealing from nor donating to the charity. What Coren wants to say is that, intuitively, despite the congruence of S’s action across worlds, S should be neither praised nor blamed for what he has done (option E). In contrast, I see no problem with saying that S can be both praised and admonished for doing E, at least where the options available (‘other than E’) are *viable* options. To illustrate what I mean, let us say that every day I neither steal from the local charity shop nor do I donate to charity. But, equally, I do not consider that there has ever been a viable opportunity for me to steal from the charity shop, nor, owing to my utter lack of disposable income (let us allow), do I consider donating to charity to be a viable option. In contrast, for the alternate possibilities described in W_2_ to be genuine possibilities, they must be viable options.

Returning to Case 3, then, suppose a member of the charity accidently left a cash donation where S could easily have stolen it undetected. In W_2_, we can allow that S had a *viable opportunity to steal* but did not. As far as the TWC is concerned, job done (so to speak), moral responsibility can be assigned on the basis of this one alternate possibility (which is the main concern of PAP_(s)_ and the TWC) and praise (in this case) administered. But, equally, S did not donate to the charity, which was another option.^5^ What concerns us, here, is whether we should be more morally obliged not to steal than we should be morally obliged to donate to charity. As I mentioned previously, I see no inconsistency in praising S for not stealing whilst also admonishing him for not donating to charity, providing each of these is a viable option.

To further illustrate how one could ascribe moral responsibility whilst also varying one’s degree of praise (say, by assigning a mixture of praise and admonishment), consider the following (final) example: Donald is a billionaire who finds himself on a TV quiz show. As part of the show, Donald has to select one box from a series of numbered boxes. Inside the box Donald selects is a card informing him that he has been awarded £1000 prize money. He is also told that he has two options: (i) keep the money, or (ii) keep half the money and give the other half to charity. Intuitively, the situation as described satisfies the requirements of PAP_(s)_ – because it contains one contextualized immoral option and one contextualized moral option – and so Donald should be ascribed moral responsibility for whichever decision he makes. In line with our intuition, he should be praised for selecting (ii) and admonished if he selects (i). Now suppose, instead, that Donald has available to him numerous options. He could (i) keep all the money, (ii) give 50% away (to charity), (iii) give 75% away, (iv) give 99% away, or (v) give it all away. Suppose Donald chooses (iv). Is (iv) *clearly* morally inferior to (v) or clearly morally superior to (iii) or (ii)? To be fair, I believe, this is a point Coren is trying to make by way of a criticism of PAP_(s)_ and even PAP_(s)_*. But, in doing so, the focus has shifted from whether moral responsibility should be ascribed to S – which remains the case even with the increase in options available – to how much S should be praised or admonished, given that he *is* morally responsible.

First and foremost, the role of PAP_(s)_, in conjunction with TWC (where necessary), is to determine whether moral responsibility should be ascribed to Donald (in this case). Typically, where Donald selects an option identified as moral he should be praised; where he selects an immoral option he should be admonished. Is it immoral for a billionaire to keep all of the £1000 lb prize money? Is it morally good for him to keep only half and give the rest to charity? Is it morally better to give away 75% and better still to give away 99%, and is the best thing for him to do, morally, to give it all away? What is required for the ascription of moral responsibility is for at least one option to be the moral inverse of whatever else is available. If one decides that keeping all of the money is immoral (although I recognize that some readers may require a defence of this claim) and if one considers more than one option to be moral, and S selects one of the moral options, then there is no inconsistency in saying that giving half of the prize money to charity is a morally good thing to do (compared to keeping all of it) and S should be praised for that, while also accepting that giving away 75% would have been better and therefore deserving of more praise, while 99% would have been better still, and giving all of it away deserves the most praise.

## Conclusion

I concede that in Young (2016) the definition of alternate possibilities was imprecise because it was focused on providing an alternate possibility to an immoral outcome. Coren is therefore correct to point this out. I consider my amended definition to be able to withstand Coren’s objections. I further concede that Coren’s amended version of PAP_(s)_ (his (PAP_(s)_*) is correct to highlight the relativity at play when comparing moral outcomes: that some are morally superior or inferior to others, and necessarily so, for the ascription of moral responsibility. Nevertheless, I consider the reasoning underlying PAP_(s)_ to endorse this relativity, even though it is not made explicit within PAP_(s)_’s formulation. Having said that, I believe that it is important to identify outcomes in moral or immoral terms, and I say this while accepted that such statuses may well be context dependent (the exception being outcomes that are unequivocally moral or immoral). Ultimately, this must be done if one is to distinguish an outcome that is *clearly* morally superior or inferior from one that is not: for it is my view that the term ‘clearly’ is marking out a different moral kind, identifiable in terms of its status as either moral or immoral relative to the context in which it occurs (again, accepting the caveat that relates to unequivocal outcomes). Finally, I consider PAP_(s)_ to be in keeping with our intuitions about being able to do otherwise; but also consider being able to do otherwise to be subtly different to necessarily drawing on alternate possibilities for the ascription of moral responsibility, which is why I consider *either* PAP_(s)_ or TWC to be necessary, rather than PAP itself. I also hope to have clarified the role congruence plays when employing the TWC. Congruence relies on outcomes being identified as moral or immoral in addition to their relative status as superior or inferior to something else.

In conclusion, then, what I hope to have done is present a defence of PAP_(s)_ and TWC while also amending these in response to Coren’s comments. My amended version of PAP_(s)_ now reads as follows:

### PAP_(s)_

Where E and ‘other than E’ have moral-aptness, S is morally responsible for doing E if S could have done ‘other than E’, where doing ‘other than E’ consists in having available at least one alternate possibility that is the moral inverse of E.

Identifying an outcome as moral or immoral does not require that the outcome is unequivocally moral (which would require that no ‘other than E’ option, whether available or not, is morally superior to E) or unequivocally immoral (which would require that no ‘other than E’ option, whether available or not, is morally inferior to E). It requires only that E and ‘other than E’ are the moral inverse of each other, *relative to* each other. Where no alternate possibility (‘other than E’) is available, as described, then PAP_(s)_ is not satisfied. S will therefore only be ascribed moral responsibility if the TWC is satisfied. The TWC has also been amended slightly, so that it now reads:

### TWC

Where E and ‘other than E’ have moral-aptness, and where S’s action E in world W_1_ (a world without alternate possibilities) and S’s action E in twin world W_2_ (which differs from W_1_ only insofar as it is a world with an alternate possibility) are congruent in terms of *moral outcome* but not necessarily in terms of *action*, then S in either world is morally responsible for E.

## Footnotes

1 To ascribe morally responsibility to S in this scenario, the Twin World Condition (TWC) would need to be taken into account (yet to be discuss here). Only if S’s action was found to be congruent in W_1_ and W_2_ (where W_2_ contains at least one option that has an inverse moral outcome to any other), should S be ascribed moral responsibility and, in this case, praised. Should S’s action in each world instead be incongruent (insofar as an immoral outcome was selected in W_2_) then S should not be ascribed moral responsibility in W_1_ nor praised in W_2_.

2 An outcome that is unequivocally immoral compared to one that is not unequivocally immoral means that there is some difference between them (they differ by some indeterminate degree; say, in terms of the *number* of alternate possibilities they are *not* morally superior to: all compared to some), but are still of the same *immoral* kind.

3 Of course, options identified as amoral in one context – e.g., choosing to eat either a croissant or a Danish pastry – may well be considered moral in a different context where a further option is immoral (e.g., eating a person).

4 There is a problem with the scenario I have just described involving Black’s intervention. What would Black do in the context of Coren’s Case 1 where (I have argued) S can only do something morally good or morally bad. Let us leave that issue aside, however, as it does not concern the point Coren is making with Case 3.

5 In Coren’s original Case 3, if in W_2_ S decided to donate to charity then presumably this meant he did not steal, although the two are not mutually exclusive. After all, one could contrive that S stole the original donation and then returned it to the charity, not as the original donation (that he could claim to have found) but as a new donation from himself (perhaps so that he would look good to others). Of course, one may discount this as a genuine donation, given that it was stolen money.
